# Hospital bed capacity across in Tunisia hospital during the first 4 waves of the COVID-19 pandemic: A descriptive analysis

**DOI:** 10.1016/j.imj.2023.04.004

**Published:** 2023-04-25

**Authors:** Slimane BenMiled, Chiraz Borgi, Mohamed Hsairi, Naoufel Somrani, Amira Kebir

**Affiliations:** a Bio-(Informatic, Mathematics and Statistic) BIMS-Lab LR09-IPT16, Institut Pasteur de Tunis, University of Tunis el Manar, Tunis 1002, Tunisia; b Ministry of Health, Tunis 1002, Tunisia; c Department of Epidemiology and Preventive Medicine, Faculty of Medecin, Tunis El Manar University, Tunis 1002, Tunisia; d IPEIT, University of Tunis, Tunis 1002, Tunisia

**Keywords:** Bed occupancy, Daily cases, Daily death, Ramp duration until the peak (RDUP), Ramp growth until the peak (RGUP), Ramp rate until the peak (RRUP)

## Abstract

**Background:**

In March 2020, the WHO declared COVID-19 as a pandemic, and Tunisia implemented a containment and targeted screening strategy. The country's public health policy has since focused on managing hospital beds.

**Methods:**

The study analyzed the bed occupancy rates in public hospitals in Tunisia during the pandemic. The evolution of daily cases and nonpharmaceutical interventions (NPI) actions undertaken by the Tunisian Government were also analyzed. The study used 3 indices to assess bed flexibility: Ramp duration until the peak, ramp growth until the peak, and ramp rate until the peak. The study also calculated the time shift at the start and peak of each wave to evaluate the government's response efficacy.

**Results:**

The study found that the evolution of the epidemic in Tunisia had 2 phases. The first phase saw the pandemic being controlled due to strong NPI actions, while the second phase saw a relaxation of measures and an increase in wave intensity. ICU bed availability followed the demand for beds, but ICU bed occupancy remained high, with a maximum of 97%. The government's response in terms of bed distribution and reallocation was slow. The study found that the most deadly wave by ICU occupied bed was the third wave due to a historical variant, while the fifth wave due to the delta variant was the most deadly in terms of cumulative death.

**Conclusions:**

The study concluded that decision-makers could use its findings to assess their response capabilities in the current pandemic and future ones. The study highlighted the importance of flexible and responsive healthcare systems in managing pandemics.

## 1 Introduction

On 30 January 2020, COVID-19 was retained by the World health organization (WHO) as a public health emergency of international concern; and WHO^1^ announced the pandemic threat on March 11, 2020, [1].

In Tunisia, the first COVID-19 wave appear on March 2, 2020, followed by several implemented preventive measures to reduce transmission levels among the population. These measures included mask use, physical distancing, school and university closures, sports and cultural events ban, borders closure, targeted screening, and finally a national lock-down announced on March 22, 2020, [2]. However, in July 2020, Nonpharmaceutical interventions (NPI) measures have been significantly reduced. This resulted in more intense waves beginning in October 2020, forcing the Tunisian Ministry of Health to respond rapidly by strengthening its resources, particularly in terms of hospital management.

In these conditions, better healthcare administration becomes a crucial concern [3]. Flexibility in hospital bed management, in particular, has become critical in treating patients with severe and/or serious COVID-19 infections [4].

The goal of this study is to describe the COVID-19 situation between July 2020 and February 2022, by using the data from the Facebook coronavirus survey^2^, Health Metric Data^3^ and using newspaper investigation. We also investigate hospital bed occupancies (beds for patients who require oxygen/ICU beds) at the national level. On the other hand, a mathematical index was developed and used to analyze the time shift between daily cases and mortality, as well as bed occupancy.

In Section 2, we go over the databases used in this study, as well as the methodology and tools used to analyze the data. We present our main results in Section 3 which we discuss in Section 4. Finally, in Section 5 a conclusion is presented.

## 2 Materials and methods

### 2.1 Data collection method

Bed occupancy are collected by public Tunisian hospitals based on data from the COVID-19 SHOC Room (Strategic Health Operations Center Room) (SR) and the Ministry of Health's Computer Centre (CIMS). The goal of the SR was to collect daily data from hospitals, communicate with them to filter and debug mistakes, and illustrate the results. The data was collected daily using a spreadsheet from 168 hospitals located throughout Tunisia's 24 regions. The CIMS is in charge of running the administrative management system for admissions and exits in hospitals with an IT management system. Specifically, the 168 hospitals in the SR database.

Collected data ranked hospital beds (i.e., beds that had all the equipment and personnel necessary for their function) according to 2 large groups, ICU beds, and non-ICU beds called here O2 beds; ICU beds are used in intensive care units (ICUs) to treat patients with serious or life-threatening illnesses and traumas. ICUs differ from ordinary hospital wards in that they have a higher staff-to-patient ratio and have access to advanced medical resources and equipment not commonly found elsewhere. The beds assigned to each group were further subdivided into allocated beds (i.e.*,* available bed to patient), occupied beds (beds that were occupied by inpatients), and unoccupied beds (i.e., available beds that were not occupied). Allocated beds are constituted by allocated and available beds.

For our analysis, we collected the following daily reported data stream: (i) The SR database contains data on O2 dedicated beds, O2 occupied beds, O2 available beds, ICU dedicated beds, ICU occupied beds and ICU available beds. (ii) The CIMS database that contains data on admissions and exits date for each COVID-19 patient, the hospital unit of entrance and exit, and its status at existing (i.e., death or not). Both SR and CIMS databases cover the period from September 2020 to the end of February 2022. The data have been cured and aggregated at the national level. Database is available on GitHub^4^.

**Fig. 1:**
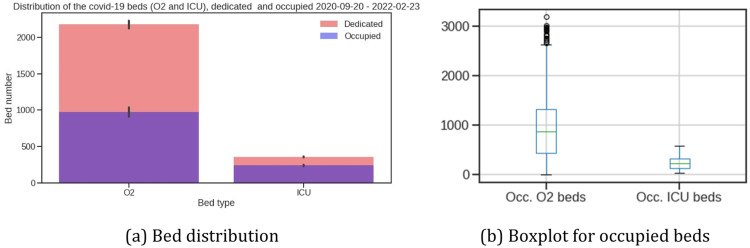
Distribution of hospital beds by bed type: O2/ICU beds dedicated/occupied.

During this period the mean occupied O2 bed was 972 and the mean occupied ICU bed was 240 (see Fig. 1 and Table 1).

**Table 1:** Average, min, and max bed occupancy.

	O2			ICU		
	Average	Min	Max	Average	Min	Max
Occupancy	42%	2%	90%	66%	10%	97%
Date		18/11/2021	07/07/2021		11/11/2021	26/07/2021
Dedicated	2,175	2,410	2,602	359	361	544
Occupaied	972	61	2,367	240	37	528

In the remainder of the document, we will use the term *bed curves* or *bed waves* for curves and waves relating to data on allocated or occupied beds ICU or O2.

Since both the epidemiological and bed occupancy data are noisy, we smoothed them using a linear convolution one dimension kernel, *Box*1*DKernel* (*N*) from *astropy.convolution* [5] library on Python 3 [6]. Following testing with several bandwidth choices, we found that *N* = 7 is the lowest that can still smooth the curve.

### 2.2 Data analysis methodology

To study the flexibility of bed occupancy, [7,8] introduced 3 distinct index: the Ramp Duration Until the Peak (RDUP), which measures the duration of the wave/bed allocation effort. The Ramp Growth Until the Peak (RGUP), which measures the peak height, and Ramp Rate Until the Peak (RRUP) which measures the growth rate of the wave (i.e., $\text{RRUP}\;=\;\frac{\text{RGUP}}{\text{RDUP}}$) (see Supplemental Fig. A.1). Where bed occupancy is defined as the ratio of occupied beds to the total number of allocated COVID-19 beds. In the case of bed allocation curves, RRUP measure the intensity of the bed allocation effort. We applied a linear convolution one dimension kernel to estimate these indices. It should be noted that [8] used the Linear Hinges Model instead.

The RDUP can be computed from the difference between the time where the peak is reached *t_p_* and the starting time of the increase of the wave (i.e., the start of the wave), *t*_0_. RDUP corresponds to the time to reach the curve peak. In the same way, the RGUP can be obtained from the difference between the number of available beds at the peak *n_p_* and at the starting time of the increase in the demand *n*_0_.

To match different waves, we also calculate the time shift or delay at the start (resp. at peak) of each 2 wave (see Supplemental Fig. A.2). This time correspond to the shift at start (resp. at peak) between 2 waves. The waves can be daily cases of bed occupancies.

Estimation the wave start and the peak. The classical definition of the start of an epidemiological wave involves the evaluation of the effective reproduction number, *R_t_*, defined as the average number of secondary cases per infectious case in a population made up of both susceptible and nonsusceptible hosts [9].

Regardless of the classical definition of the effective reproduction number, *R_t_*, its utility stems from the fact that it reveals the curve's exponential growth. To be more specific, if *R_t_ >* 1, the curve grows exponentially, whereas if *R_t_ <* 1, the curve shrinks to zero. As a result, the waves beginning and peak correspond to the time when *R_t_* = 1. [10].

Thus, we estimate the start of a wave as the time, *t*_0_, when *R_t_*_0_ = 1 while increasing in a neighbourhood of *t*_0_. Similarly the peak corresponds to the time, *t_p_*, for which *R_tp_* = 1 while decreasing in a neighbourhood of *t_p_*.

When applied to bed occupancy data, similarly to an epidemiological curve, *R_t_* evaluates the exponential growth of the bed occupancy data curve. To avoid any misunderstanding, we shall refer to it as a “gradient like index,” or *G_t_*. Therefore, we define the wave period using *R_t_* or *G_t_* depending on the data type, daily cases or bed occupancy. We computed *R_t_* and *G_t_* using [11].

## 3 Mathematical modelling

We adapted the CoMo model [12] to simulate the spread of SARS-CoV2 in the context of NPIs measures and vaccination strategies. The CoMo model is a dynamic SEIRS (Susceptible-Exposed-Infected-Recovered-Susceptible) model. It is an age-structured SEIRS model with infected compartments stratified by symptoms, severity, treatment-seeking, and hospital access (see Fig. A.3).

The description of the variables used and a list with all parameters included in the full model are given in the Supplementary material.

The CoMo model was adapted to the Tunisian context using daily cases and mortality data, demographic data from the National Agency for Statistics of Tunisia^5^, information on the different types of NPI intervention measures carried out (e.g., school closer, social distance, international travel ban, mask-wearing, ...) and parameters on hospitalizations and vaccination in Tunisia (see Supplementary materials).

NPI measures were collected from Facebook coronavirus survey, Health Metric Data, and using newspaper investigation.

For further exploration of NPI effectiveness, we compared the timeline of NPI implementation with an external data source, the Oxford COVID19 Government Response Stringency index^6^^,^^7^, a quantitative measure of the strictness of government policies regulating population behaviour.

Daily cases and deaths were collected from the Official COVID-19 report from WHO database^8^.

The model was calibrated using the epidemic data in Tunisia between September 15, 2020, and February 15, 2022, and tested through June 2022. Parameters that could not be evaluated were estimated by the optimal model fit to epidemiological data.

## 4 Results

### 4.1 Reconstruction of the epidemic history in Tunisia

**Fig. 2:**
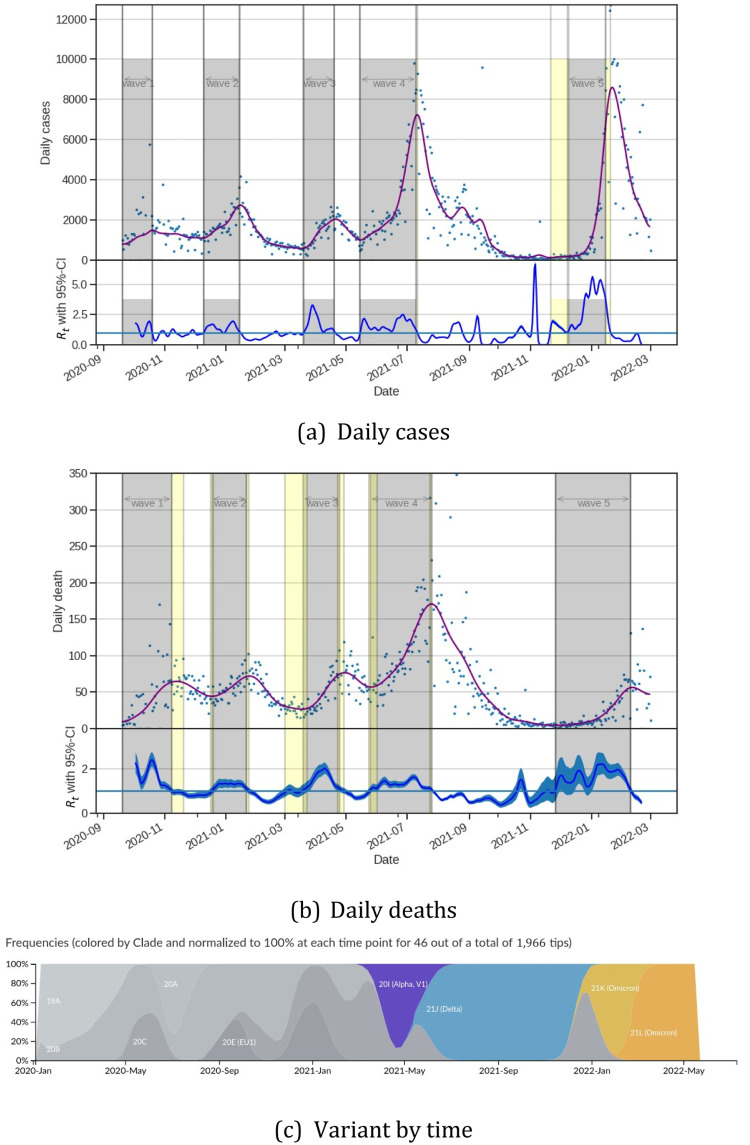
Daily cases, daily deaths with their corresponding *R_t_* and variant proportion from Mars 2019 to February 2022. The grey areas correspond to the different waves. We observe, that the waves have been more and more intens. Variant figure taken from nextstrain.org/sars-cov-2 [13].

From March 2020 to February 2022, Tunisia had 6 pandemic waves. The first one was held from March to June 2020, the second from September to October 2020, and the third from December to January 2021. The historical variant was the most common virus during the first 3 waves. The alpha variant caused the fourth wave, which occurred between March and May. The delta variant generated the fifth wave, which occurred between July and August. The Omicron variant produced the last wave, which lasted from December 2021 to January 2022 (see Fig. 2 and supplementary database) 9. These 6 waves were becoming increasingly large.

By March 28, 2022, a total of 1*,*033*,*731 confirmed cases and 28*,*165 deaths due to COVID-19 were recorded.

**Fig. 3:**
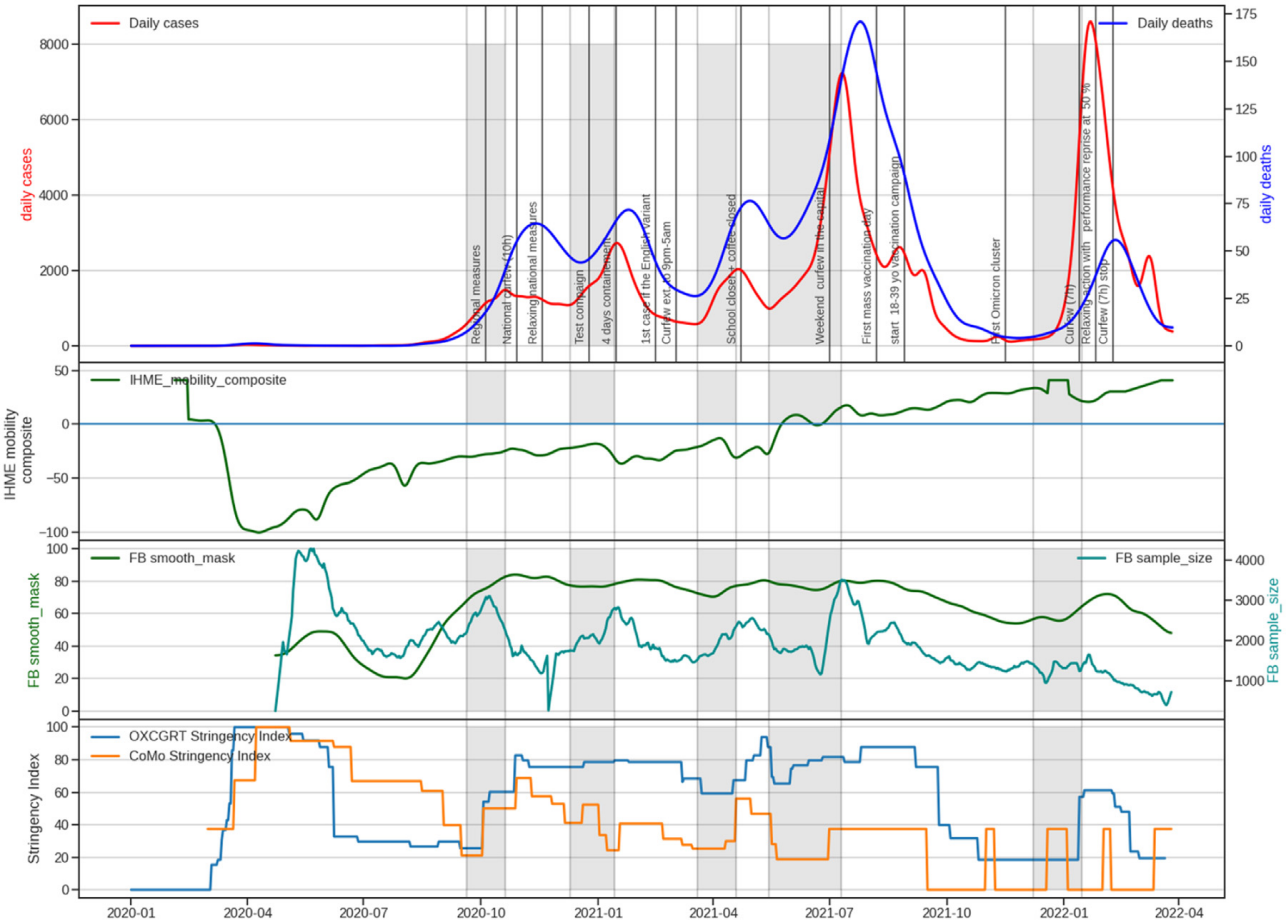
Evolution of COVID-19 in Tunisia. From the top to buttom: (1) Daily confirmed case incidence across Tunisia. (2) The IHME mobility index. (3) Facebook coronavirus mask wearing survey. (4) The Oxford NPI stringency index and stringency index calculate with NPI values used in the CoMo model. We observe, that the NPI measures have been less and less followed despite strong NPI decisions.

The Oxford Stringency Index showed the first phase of growth from March to June 2020, followed by a second phase from October 2020 (see Fig. 3). Since then, the Oxford Stringency Index has been rather stable, ranging between 60% and 100% during school holidays. Furthermore, except for the first and second waves, no variation in the stringency index occurs before the epidemic's peak. For example, during the second wave, a 12-hour curfew was imposed at the peak. The decline of this second wave appears to have been caused by regional NPI efforts between September and October 2020, as well as an increase in mask-wearing (see Fig. 3).

To understand the response to the NPI actions, we plotted IHME mobility composite between October 2020 and February 2022 (see Fig. 3). We observe that after a decrease in the mobility index between March and September 2020, the mobility index began to increase and turn to be positive from May 2021. From that date on, people's mobility was higher than in previous years. Therefore, NPI decisions undertaken by the government to limit movement had a limited effect, and even from May 2021, these decisions had almost no effect.

During the second wave, between August and October 2020, the maskwearing curve shows a significant increase. It was followed by a period of little change in the values (*<*20%) until September 2021. During the start of the wave, we observe an increase in both mask use and the number of responses to the inquiry. It is worth noting that the number of participants in the questionnaire has decreased significantly since the fourth wave, which could skew the results starting in July 2021.

To better understand the effect of the NPI actions taken, we simulated them using the CoMo model. We then evaluated the outcome of the NPI measures by minimizing the model output with the observed epidemiological data (e.g., daily cases and daily deaths). The simulation of the daily cases and daily deaths showed that the model accurately depicts the observed data (see Fig. A3).

We then calculated the Oxford Stringency Index on the NPI action used to calibrate the model (Fig. 3).

### 4.2 Bed occupancy analysis

From March 2020 to February 2022, the average occupancy for the ICU beds was 75% (273 occupied beds) and the average occupancy for the O2 bed was 50% (1,134 occupied beds) (see Table 1). The maximum occupancy occurred on July 26, 2021, with 97% occupancy of ICU beds and 90% for O2 beds on July 7, 2021.

**Fig. 4:**
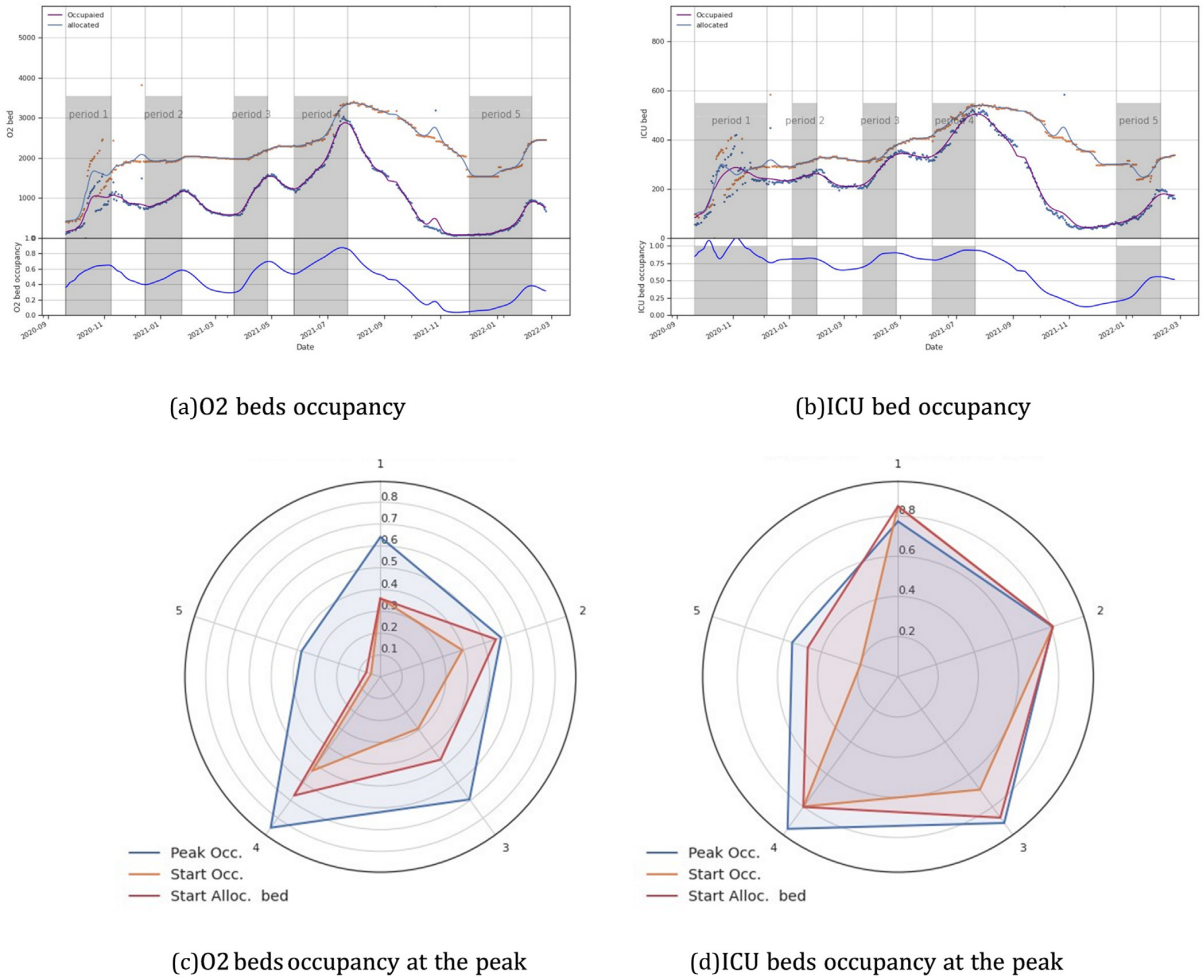
Bed occupancies for O2 beds (a, c) and ICU beds (b, d) from March 2020 to February 2022. We observe 6 waves for O2 and ICU beds of various intensities (a, b). There is very small difference between occupancy at peak and at the beginning of the each wave for the ICU bed.

Similarly to the epidemiological curves, 6 waves were seen for O2 and ICU beds of various intensities (see Fig. 4A, B). Note that, for dedicated beds, the third and fourth waves were nearly identical. Moreover, until August, the number of dedicated beds continued to increase and did not change as much as the occupied bed curves. These 2 events highlight the government's continued attempts to distribute beds.

The occupancy of ICU beds shows little change and stays near to 80% except for the third and fourth waves, when it exceeds 90%, indicating that ICU services remained under pressure during the 4 first waves (see Fig. 4D), even though it has been able to adapt to the increasing severity of the epidemic.

In contrast to the ICU bed, the O2 bed occupancy curve oscillates and ranged around 50%, except for the third and notably the fourth waves, where it reached a maximum of 90% on July 7, 2020 (see Fig. 4C).

We observe that occupancy at the start of the occupied bed wave was less than at the start of the dedicated bed for O2 beds. It was, however, roughly equal for ICU beds (see Fig. 4D). This discrepancy may be explained by the fact that ICU bed distribution during waves is based on demand with a quick response.

This observation is also supported by an evaluation of the time lag between the start of the waves of occupied and dedicated beds. Indeed, we find that the average time shift for ICU beds is 11*.*4 days (standard deviation = 15*.*7), with a maximum of 31 days for the wave 5. The reason for the long time shift is that the rate of bed growth in wave 5 was slow (see Fig. 7D).

The average time shift between occupied and allocated O2 beds was 22*.*75 days (standard deviation = 7*.*5), with a maximum of 33 days for wave 2.

**Fig. 5:**
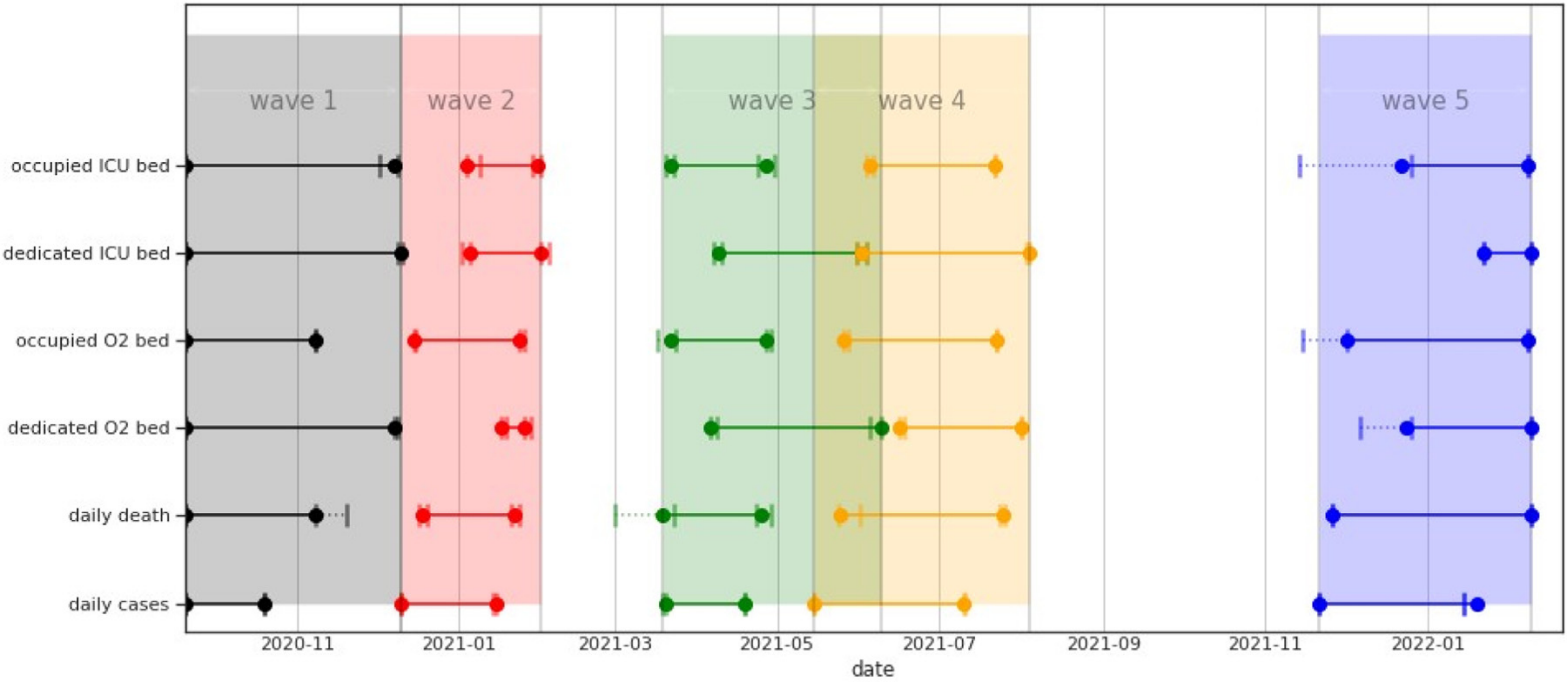
Time distribution of the different waves for daily cases, daily deaths and occupied and dedicated beds.

**Fig. 6:**
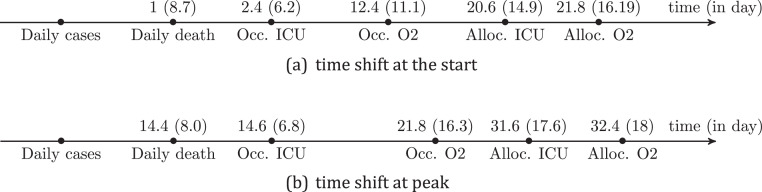
Mean shift and std at the start the peak by waves for for daily death, allocated and dedicated O2 and ICU beds. The shift is calculated relatively to daily cases curves. There is a shift in the timing of the peak of bed occupancy waves curves in relation to the daily cases wave. Furthermore, beds are still being allocated after the peak of occupancy.

We also plotted the occupancy at the start of each wave, and for O2 and ICU beds (see Fig. 4C, D). We found that the occupancy at the start of the allocated bed waves was higher than the occupancy at the start of the occupied bed waves, especially for O2 beds. This demonstrates a time shift in bed re-allocation, particularly for O2 beds (see Figs. 5 and 6A). However, the difference in occupancy at the start of the 2 ICU bed waves (allocated and occupied beds) is small, except for the third wave, where it is 70% *versus* 90%.

We then analysed the difference in the peak times (see Figs. 5 and 6B). The order of appearance of the peak waves appears to be as follows: daily case, daily death (with an average time shift of 14*.*4 days, std = 8), occupied ICU beds (with an average time shift of 14*.*6 days, std = 6*.*8), occupied O2 (with an average time shift of 121*.*8 days, std = 16*.*3), dedicated ICU bed (with an average time shift of 31*.*6 days, std = 17) It should be highlighted that the difference between daily death and ICU occupied, as well as dedicated beds, is extremely small (see Fig. 6B).

Furthermore, it appears that beds are still being allocated after the peak of occupancy, which lasts an average of 10*.*6 days (std = 15) for ICU beds and 17 days (std = 18*.*6) for O2 beds. The maximum time shift for ICU was observed for wave 3 where the time shift was 36 days at the pick, For O2 bed, we found that during wave 3, O2 beds continue to be allocated 43 days after the peak of the occupied bed's wave (see Fig. 6B).

For all waves, we observe that the peak of deaths occurs before the peak of ICU bed occupancy. This mismatch might be caused by patient bed occupancy periods or by a carry-over of deaths from the previous wave to the beginning of the new wave. However, the mean time spent in ICU is 7*.*33 days (ssd = 5*.*88) for dead and 6*.*83 days (ssd = 5*.*45) for recovery and therefore, cannot explain this discrepancy. An alternative could be the large values of standard deviation, 5*.*88 days for death and 5*.*45 days for recovery. Another explanation could be the high number of death occurring outside the hospital.

To investigate the duration and strength of each wave, we studied the evolution of the ramp duration until the peak (RDUP) and the ramp rate until the peak (RRUP).

**Fig. 7:**
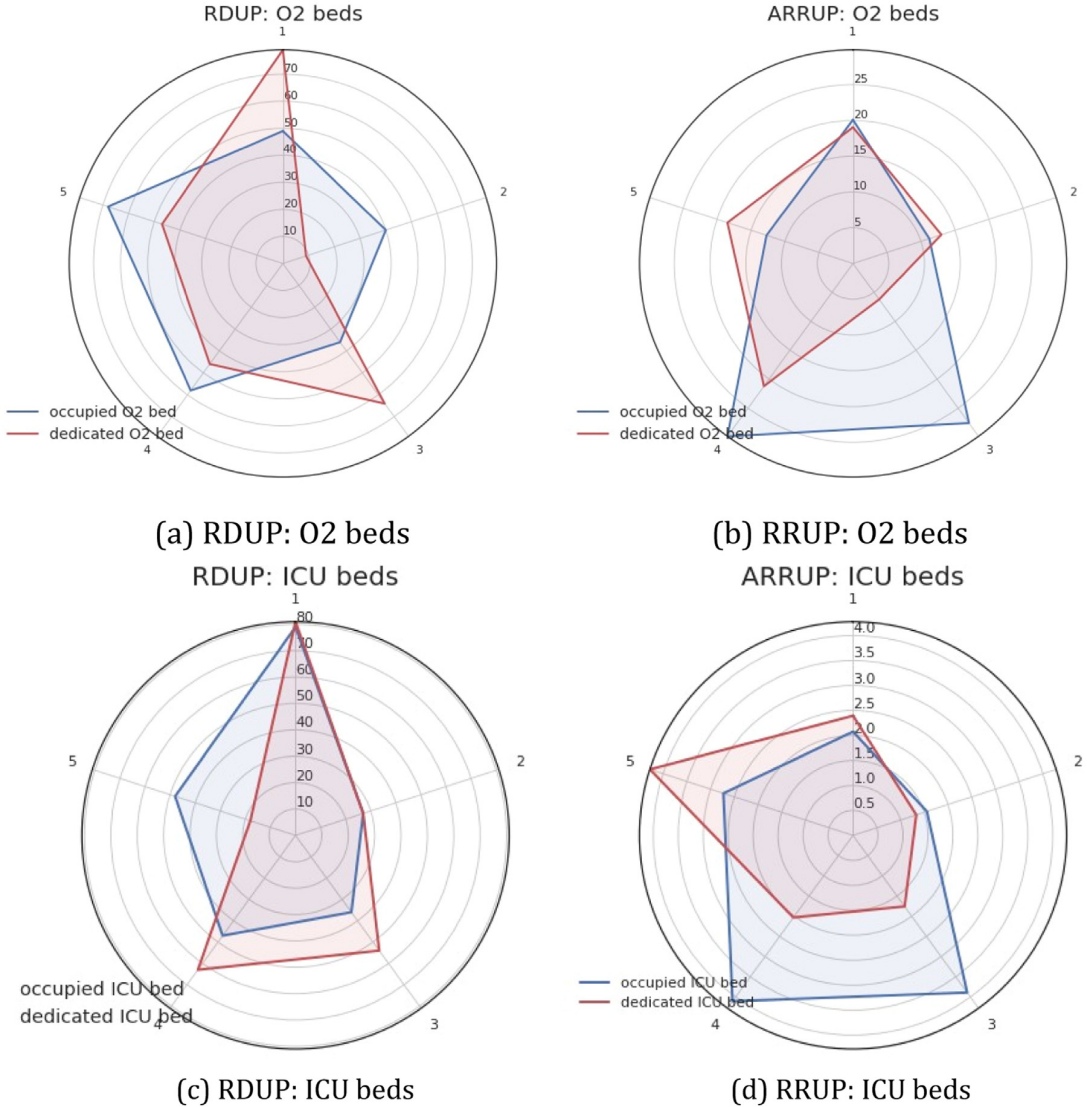
Ramp duration until the peak (RDUP) and ramp rate until the peak (RRUP) for the five waves. We observe that waves 3 and 4 were the most intense for occupied beds (b, d). On the other hand, the waves of the allocated beds were longer but less intense (a, c).

Except for waves 1 and 3 for O2 beds, the RDUP for beds occupied was longer than the allocated beds (see Fig. 7). Furthermore, except for wave 5, we found the RRUP was higher for occupied beds.

In the example of the occupied O2 bed, we can see that it rapidly increases during wave 4 (RDUP = 58 days and a massive RRUP = 29*.*8). Furthermore, wave 3 exhibits rapid growth (RRUP = 27*.*5) during a short length of time (RDUP = 36 days). The dedicated bed, on the other hand, has developed slowly and gradually.

For ICU beds, occupied has had rapid growth in a short time. Dedicated beds, on the other hand, display slower growth over a longer period (except for wave 5). The higher increase was done for wave 3 and for where RRUP = 4*.*1 (RDUP = 47 days) for wave 4 and 3*.*8 for wave 3 (RDUP = 36 days).

In a short time, the number of ICU beds occupied has increased rapidly. Dedicated beds, on the other hand, exhibit slower growth over a longer length of time (except for wave 5). The larger increase was performed for wave 3 where RRUP = 4*.*1 (RDUP = 47 days) and for wave 4 where RRUP = 3*.*8 (RDUP = 36 days).

## 5 Discussion

The analysis of COVID-19 healthcare data has revealed an opportunity to better understand healthcare performance in stressful circumstances. We were able to assess the overall responsiveness of the Tunisian public hospital system by assessing flexibility and bed margin. Decision makers will be able to assess their response capabilities in the case of a current pandemic thanks to this research.

Decision-makers in public health have to respond, especially in emergencies where the system is forced to manage critical and urgent risk [3]. This is accomplished through the proactive deployment of NPI measures such as curfews, lockdowns, masking requirements, or school and administrative closures to avoid or lessen dangers. These decisions are frequently influenced by economical restrictions. For example, during the first wave in Tunisia between March and June 2020, rigorous confinement for 3 weeks was implemented making the the number of cases to decrease. However, these measures resulted in a drop of at least 4*.*4 percent of GDP and a 21*.*6 percent unemployment rate^10^.

Economic constraints force LMIC, such as Tunisia, to choose between 2 approaches to crisis management: an economically costly strategy targeted at minimizing deaths or a policy aimed at avoiding excess deaths owing to health-system saturation.

Tunisia public health policy, beginning from July 2020, seems to be characterized only by incentives aimed at avoiding hospital overcrowding. Apart from school closure decisions, we found in our study that required NPI decisions were poorly implemented and frequently shifted. During the same period, we noticed that people were aware of the severity of the epidemic by taking precautionary measures such as wearing masks. This was most clear during the second wave, between September and October 2020, when the decision to implement a nationwide lockdown was made after the epidemiological peak. During this same period, we observed a significant increase in the use of masks among individuals.

During a health crisis, the decision-maker must determine when, how much, and where to commit the necessary resources to manage the epidemicresources are either human (human resources reallocated from one service to another) or material (bed allocations to COVID-19). The choice is additionally hampered by the lack of knowledge on (i) the current and future state of the epidemic, and (ii) the health system's reactivity in terms of the time shift in executing the remedies decided upon Tabuteau [14]. We found a negative time shift between occupancy and assigned beds at the start of the waves, as well as a positive time shift at the peak. This implies that the authorities kept allocating beds long after the peak of the occupied bed wave had passed. These time shifts were most obvious for O2 beds and during peak times. This sustained effort contributed to reducing pressure during the second and, especially, fourth waves, when the rate of increase in O2 beds and ICU was substantially slower than the rate of increase in daily patients. Furthermore, the overlap in allocated beds between the third and fourth waves may be explained by the fact that these 2 epidemiological waves were close together, with the third wave's peak of daily cases on April 19 and the start of the fourth wave on May 15.

Moreover, we observe that the higher occupancy of ICU beds at the beginning of each wave has likely resulted in a quicker rate of ICU bed allocation (mean 4*.*0 days, std = 9*.*48) compared to O2 beds O2 beds (mean 17*.*25 days, std = 13*.*72). This discrepancy in time shift might be explained by the fact that decisions were made primarily based on bed occupancy rather than a wave start evaluation. Such time shifts could be the result of a precautionary principle based on a lack of information or trust in data on the evolution of the epidemic.

We point out that S´anchez-u´beda et al. [8], have performed a similar analysis to ours. They were interested in the first COVID-19 on all the hospitals in the region of Madrid, where they evaluated the pressure observed on the hospitals. Their results show that the Madrid hospitals had to manage a massive arrival of patients during this first wave. This massive arrival was also observed in Tunisia during all waves of the study.

A limitation of our work is the absence in the database of more descriptive variables such as the age of patients by hospital ward as the number of patients in paediatric.

As a perspective, the information presented here could be useful in comparing how effective different health care systems are at responding to pandemics in terms of response time, particularly between developed and developing countries.

## 6 Conclusions

By comparing the NPI activities performed and the response in terms of daily detection cases, we were able to recreate the history of the evolution of the epidemic in Tunisia. We discovered that, except for the first wave, the NPI activities were poorly implemented, despite the importance of the decisions made. This failure to follow the constraints, along with the presence of increasingly contagious variations, allowed for the creation of great number of death.

It has been observed that the ICU bed has always been under pressure. In fact, it has been found that there is very small difference between occupancy at peak and at the beginning of the each wave for the ICU bed.

The analysis of bed occupancy revealed that there was a shift in the allocation of new beds at the start of the pandemic, as well as in the reallocation of beds at the peak. At its peak, this shift was more than 30 days on average for O2 and ICU allotted beds. These shifts demonstrate the health system's slow response to the epidemic's progression at the start of the waves and prudence at the peak.

We observed that waves 3 and 4 were the most intense in term of occupied beds. However, wave 3 was the most important in term of death by occupied ICU bed.

## Funding

This work is based on research funded in a part by the French Ministry for Europe and Foreign Affairs via the project REPAIR COVID-19 Africa coordinated by the Pasteur International Network association. The findings and conclusions contained within are those of the authors and do not necessarily reflect positions or policies of the founders. AK and SBM are part of the Vaccine Impact Modelling Consortium.

## Authors Contribution

S.B.M.: Conceptualization, methodology, data curation, validation and writing...original draft preparation; S.B.M., A.K.: formal analysis, investigation and visualization; C.B., N.S.: data provider; S.B.M., M.H., A.K.: writing...review and editing. All authors have read and agreed to the published version of the manuscript.

## Acknowledgements

The authors would like to thank both reviewers and the editor for their valuable comments that increased its readability.

## Declaration of Competing Interest

The authors declare that they have no known competing financial interests or personal relationships that could have appeared to influence the work reported in this paper.

## Data availability

Database is available on GitHub https://github.com/slimane66/bedOccupancyTunisia.git. All authors had full access to all the data in the study and had final responsibility for the decision to submit for publication.

## Appendix Supplementary materials

Image, application 1

## Footnotes

1 https://www.who.int/director-general/speeches/detail/who-director-general-s-opening-remarks-at-the-media-briefing-on-covid-19-11-march-2020.

2 https://dataforgood.facebook.com/.

3 https://www.healthdata.org/covid/.

4 https://github.com/slimane66/bedOccupancyTunisia.git.

5 http://www.ins.tn/.

6 https://www.bsg.ox.ac.uk/research/covid-19-government-response-tracker.

7 https://github.com/OxCGRT/covid-policy-tracker.git.

8 https://covid19.who.int/.

9 https://nextstrain.org/ncov/.

10 Impact Economique du COVID 19 en Tunisie. Technical report, R´epublique Tunisienne, PNUD, 2020. http://www.mdici.gov.tn/impact-economique-de-la-pandemie-du-covid-19-sur-leconomie-tunisienne-pour-lannee-2020/.
