# Supplementary material for: Hospital bed capacity across in Tunisia hospital during the first 4 waves of the COVID-19 pandemic: A descriptive analysis
Source: Infect Med (Beijing). 2023 Apr 25;2(2):112–21. doi: 10.1016/j.imj.2023.04.004 (PMC10204889; doi:10.1016/j.imj.2023.04.004)
Supplement: Supplementary file 1 [file mmc1.docx]

# Appendix A. Supplementary material


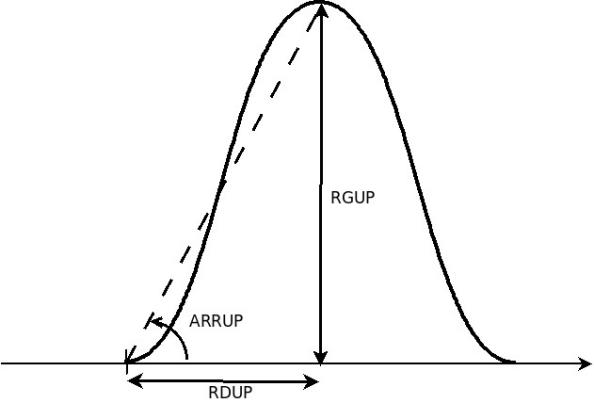


Figure A1: Flexibility index description: Ramp Duration Until the Peak (RDUP), Ramp Growth Until the Peak (RGUP) and the Ramp Rate Until the Peak (RRUP).


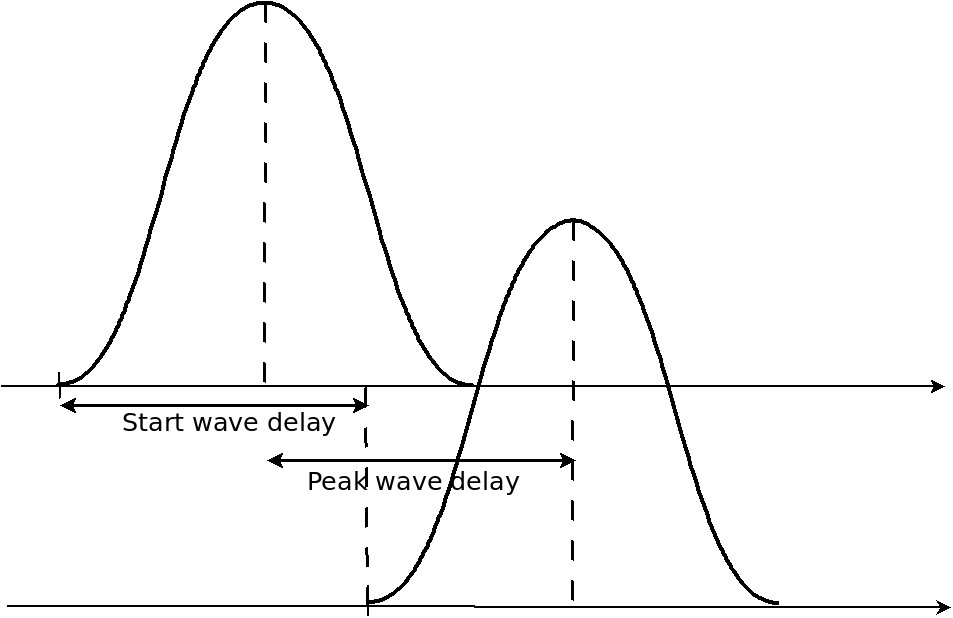


Figure A2: time shift between two waves.


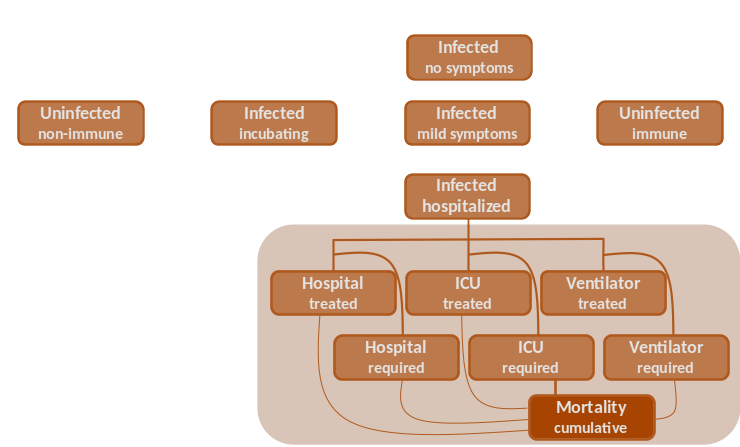


Figure A3: Conceptual view of the CoMo Model.


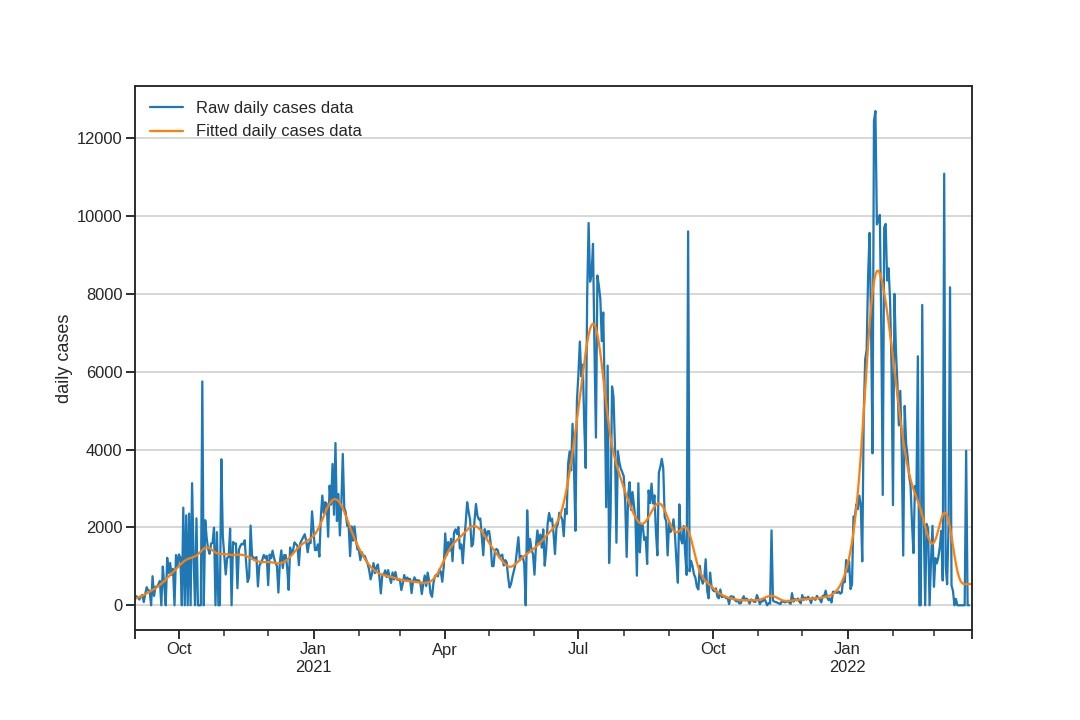


Figure A4: Daily cases: data and fitted curve. Data was fitted using a linear convolution one dimension kernel, *Box*1*DKernel*(*N*) with *N* = 7 days.


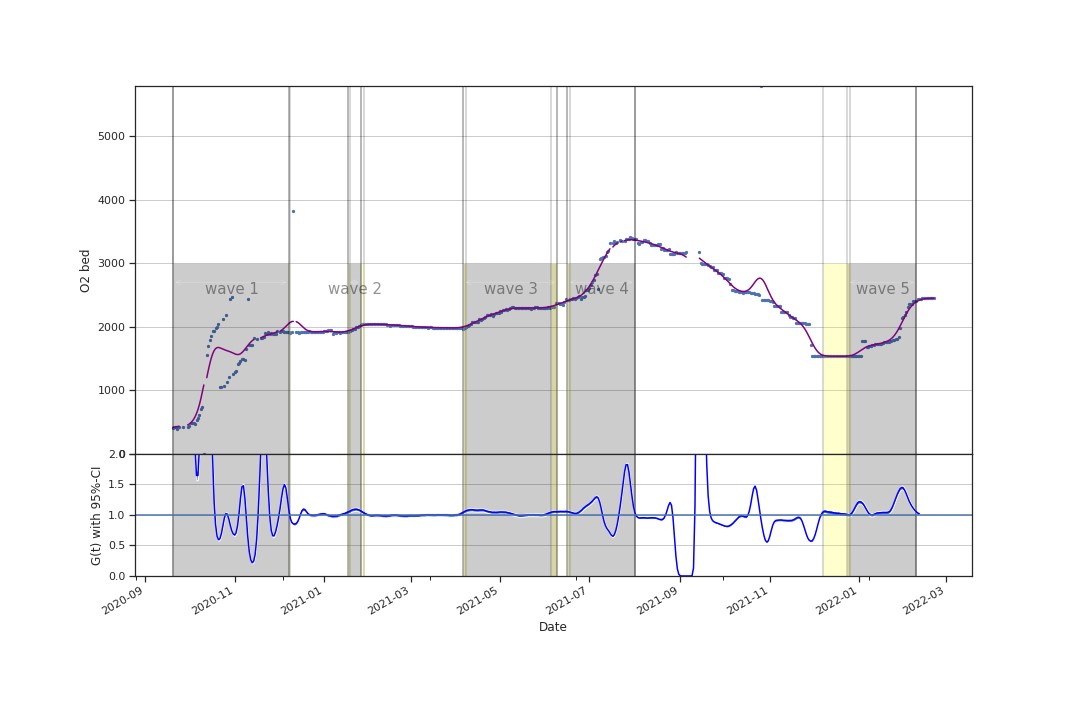


(

a)AllocatedO2beds


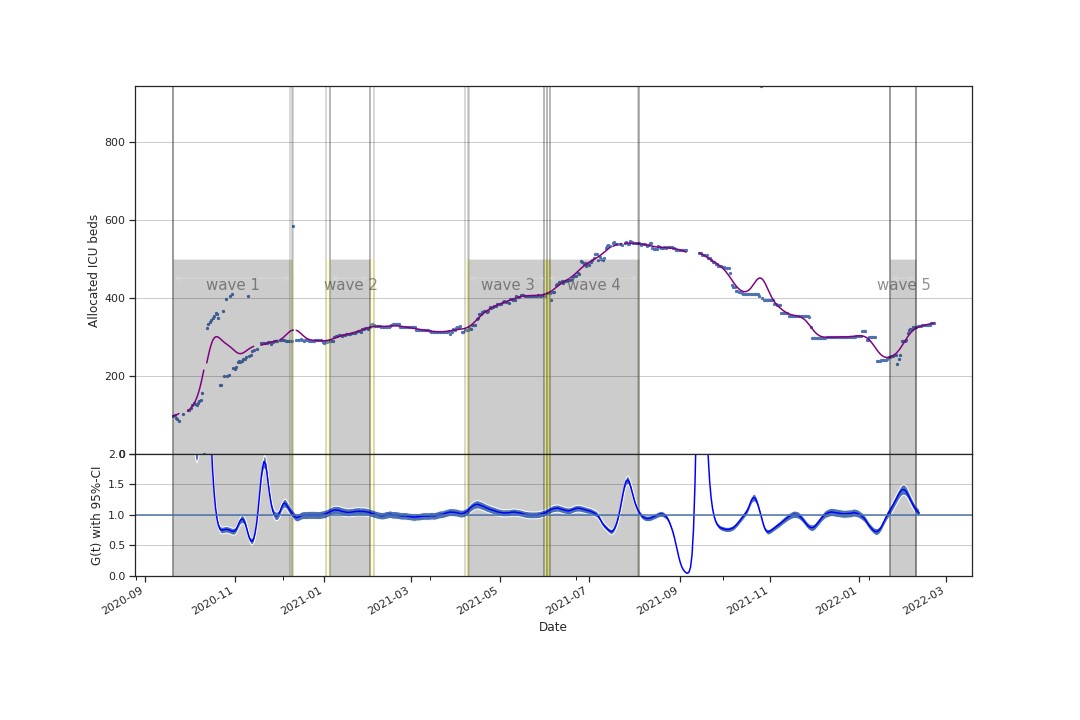


(

b)AllocatedICUbeds


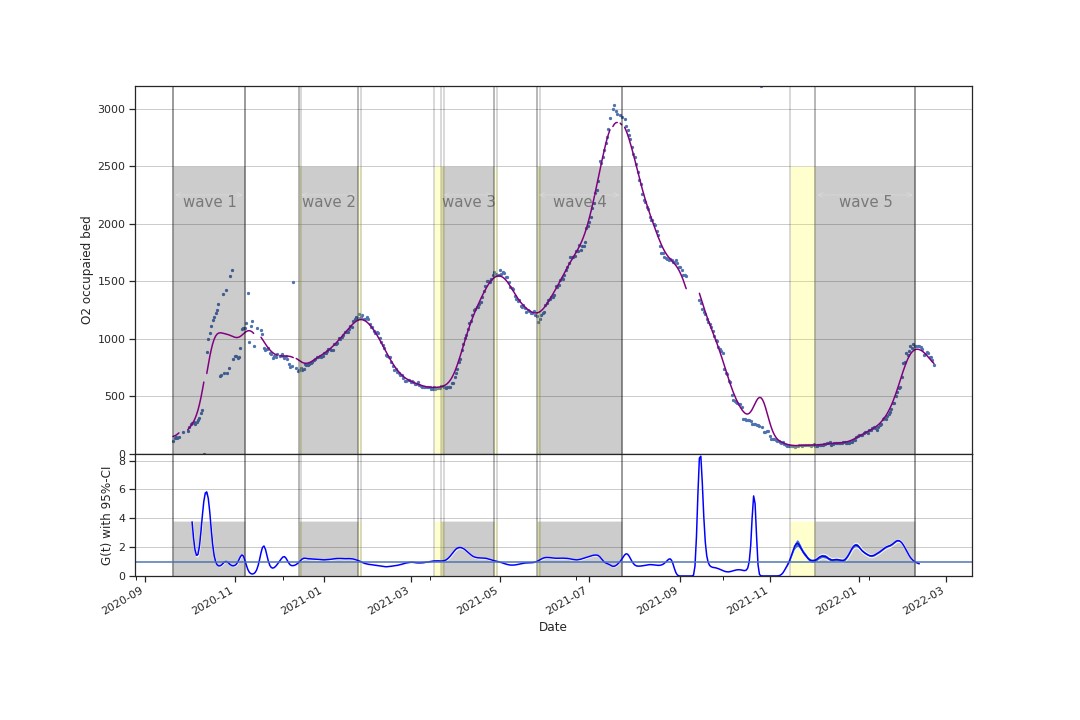


(

c)OccupaiedO2beds


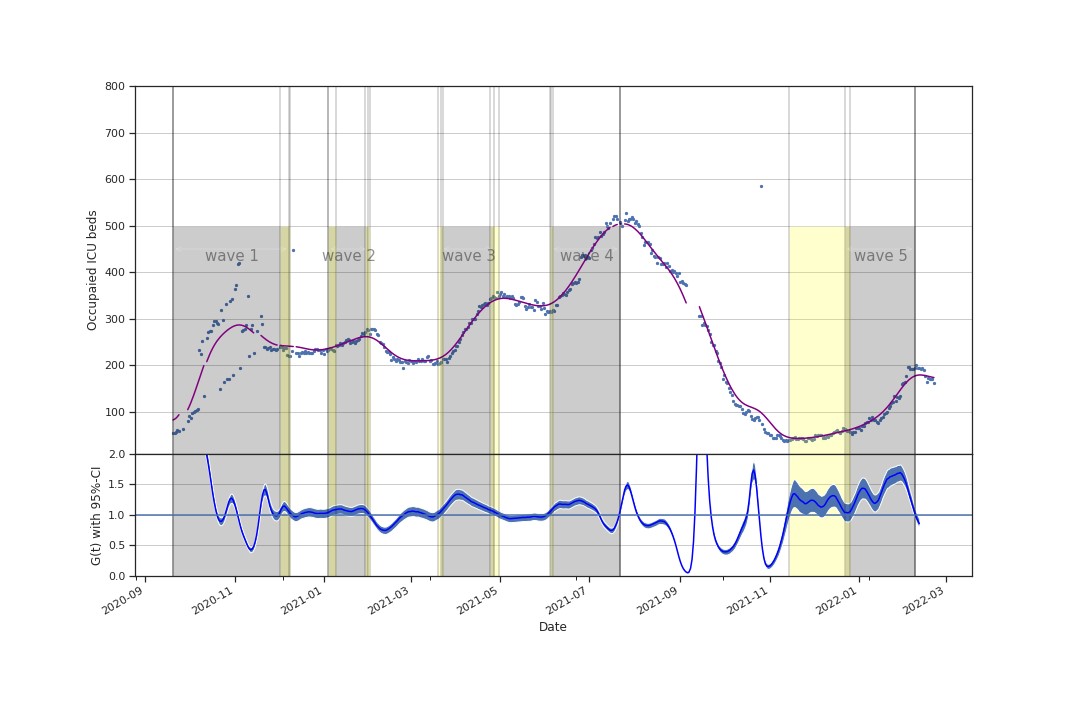


(

d)OccupaiedICUbeds

FigureA.12:Bedsevolutionbytimewiththeircorresponding

*G*

*t*

.


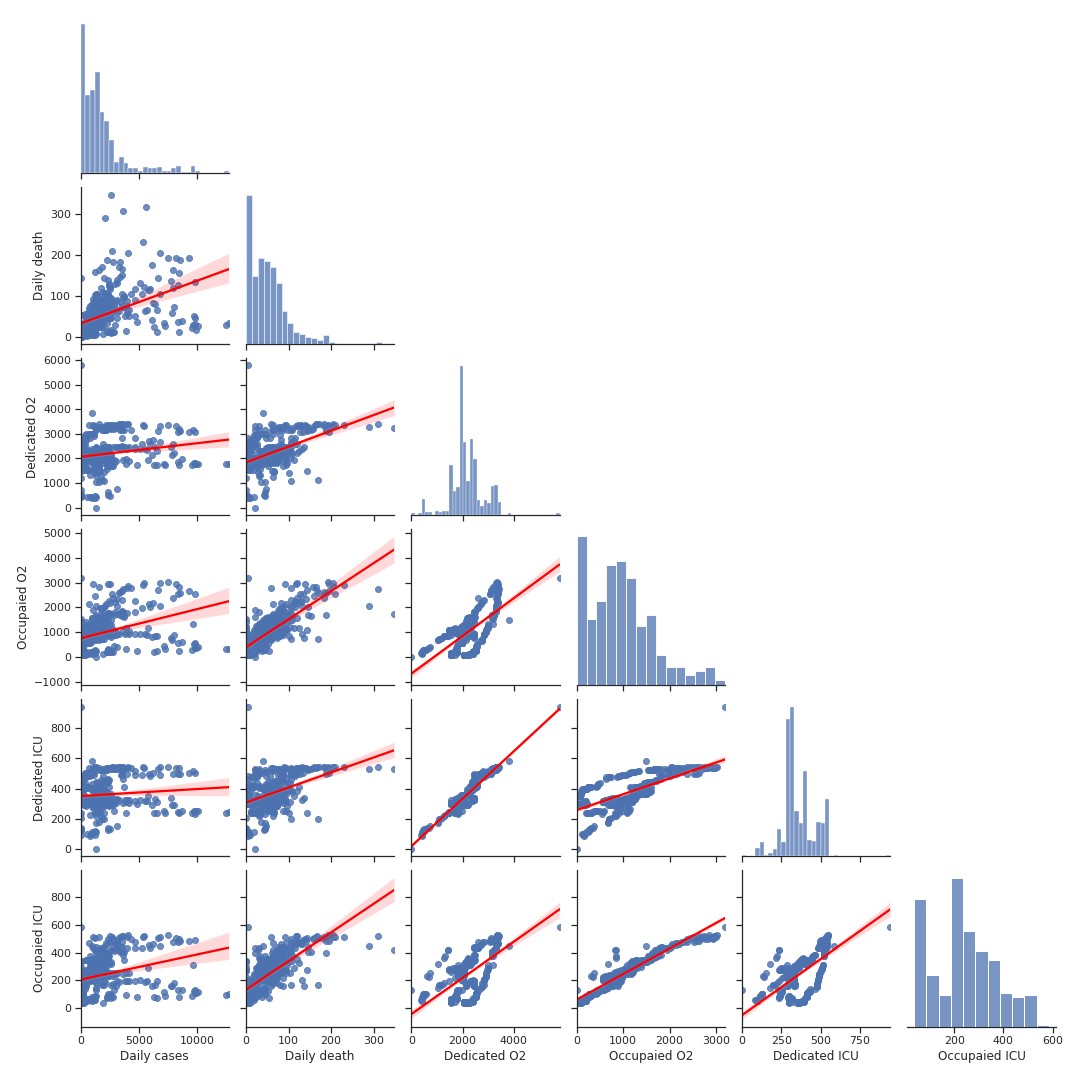


a)Linearregression

(


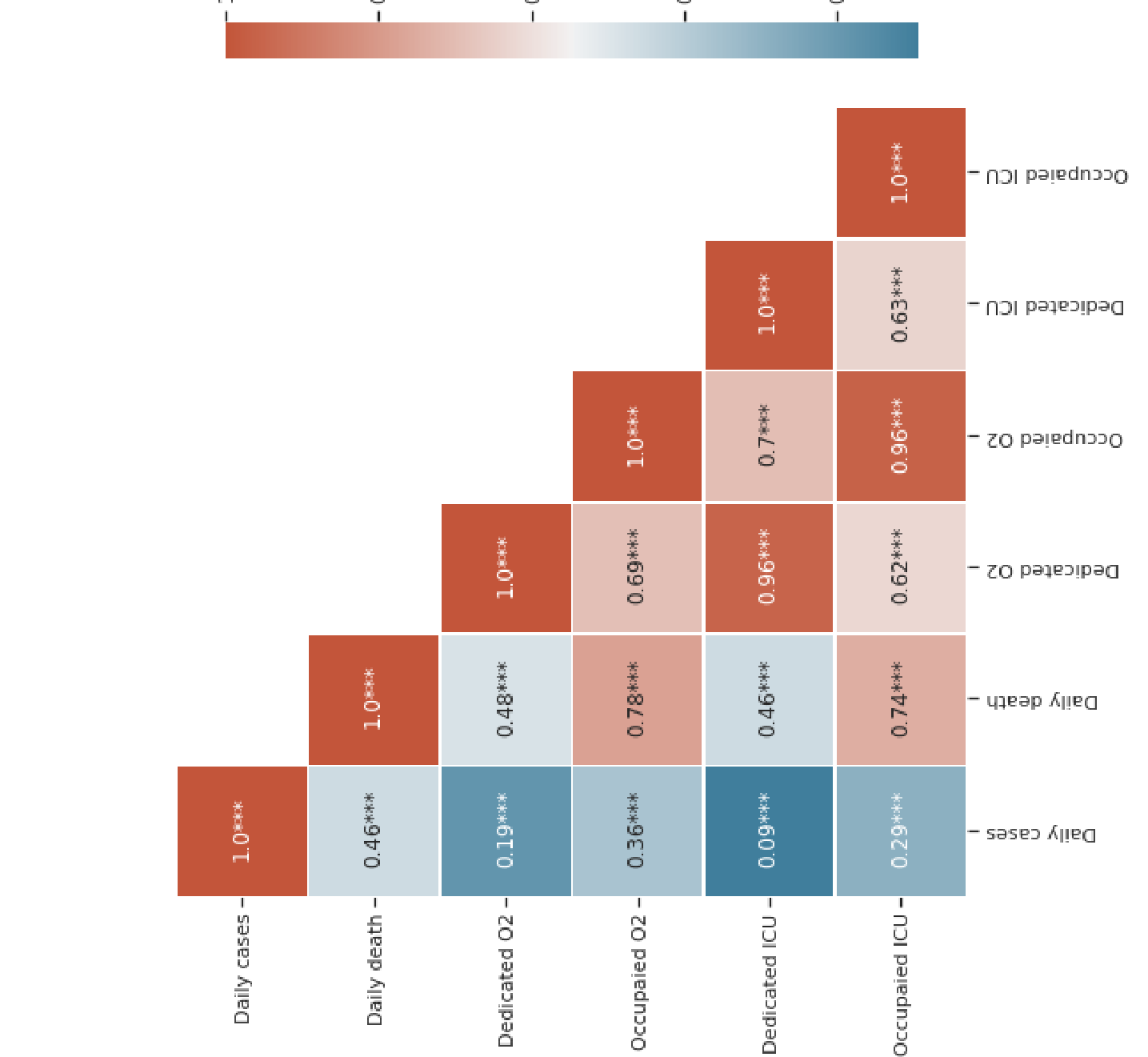


(

b)Pearsonindex

FigureA.13:PairplotandPearsonindex(***standfor

*p*

value

-

*<*

0

*.*

forbedoccupancyandepidemiologicalcurve.It

001)

appearsthatoccupiedanddedicatedbedsarestronglycorrelated(

*R*

2

*>*

0

*.*

,

8

*p*

−

*value<*

0

*.*

.Moreover,occupiedbedsare

001)

stronglycorrelatedtodailycasesanddailydeaths.However,dedicatedbedalesscorrelatedtodailycasesanddailydeaths

(

*R*

2

*<*

0

*.*

,

6

*p*

−

*value<*

0

*.*

001).

]


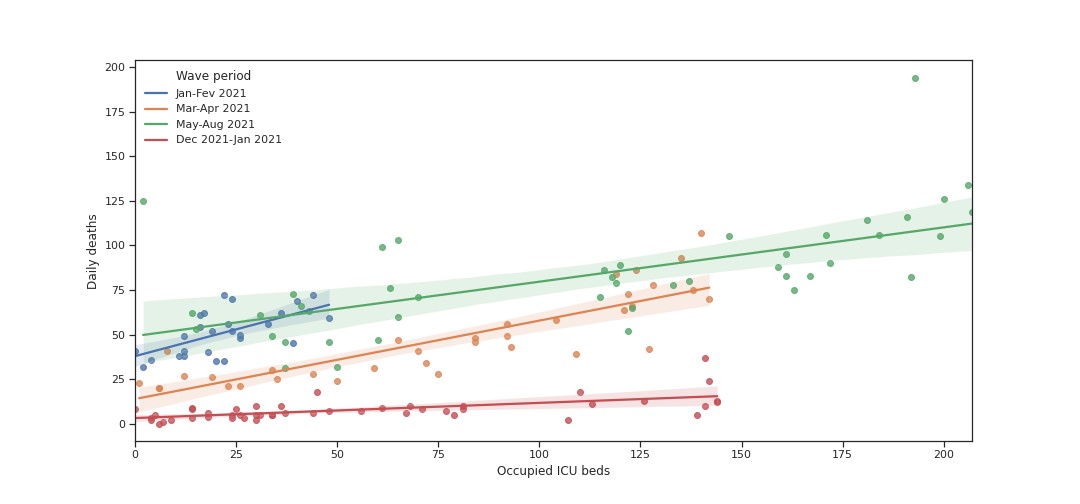


Figure A7: Linear regression between ICU bed occupancies and daily death for wave 2,3,4 and 5. The intercept is respectively 0*.*56^∗∗^0*.*42^∗∗^*,*0*.*33^∗∗^*,*0*.*08^∗∗^ (*R*2 = 0*.*34*,*0*.*74*,*0*.*465*,*0*.*387). The most deadly wave by ICU occupied bed is the wave 3 due to historical variant.
